# MEDICAID MANAGED LONG-TERM CARE PROGRAMS VS. THE TRADITIONAL MODEL: COMPARATIVE COSTS AND OUTCOMES

**DOI:** 10.1093/geroni/igz038.846

**Published:** 2019-11-08

**Authors:** Larry Polivka

**Affiliations:** Florida State University, Tallahassee, Florida, United States

## Abstract

Several states have adopted Medicaid Managed Long-Term Care (MLTC) programs over the last several years. At this point at least 30 states are either administering such models or have plans to in the near future. We do not, however, know much yet about the relative cost-effectiveness of the MLTC model when compared to the traditional non-profit model of Medicaid LTC. Is the for-profit MLTC model actually generating savings in the Medicaid program while improving the quality of care? This symposia is designed to address the question through three presentations on experiences with MLTC programs in the states of Ohio, Texas and Pennsylvania and a fourth presentation offering a national overview and critique of Medicaid MLTC in comparison to the traditional Medicaid LTC program still administered through non-profit Aging Network organizations. The state focused presentations describe the current status and results of MLTC in three states that vary in their specific features, extent of formal accountability for outcomes and the political contexts in which the programs currently function. The presentations also include discussions of the implications of each states experiences for the future of Medicaid LTC policy at the state and federal levels. The fourth presentation is a critical analysis of the main differences between the traditional non-profit model of Medicaid LTC services and the for-profit MLTC programs in terms of commonly accepted criteria of cost-effective LTC services, such as access, quality of care and per-person costs and differences in the roles of advocacy and accountability.

